# Prognostic Value of Intratumoral Expression of Claudin 18.2 in Resected Pancreatic Cancer

**DOI:** 10.1245/s10434-026-20191-5

**Published:** 2026-07-14

**Authors:** Jun Yoshino, Hakon Blomstrand, Sebastian Fjellander, Johan Svensson, Asif Halimi, Ella Kesti, Hanna Seppänen, Caroline Vilhav, Daniel Öhlund, Caj Haglund, Nils O. Elander, Peter Naredi, Malin Sund, Oskar Franklin

**Affiliations:** 1 Department of Diagnostics and Intervention, Surgery, Umeå University, Umeå, Sweden; 2 Department of Hepatobiliary and Pancreatic Surgery, Graduate School of Medicine, Institute of Science Tokyo, Tokyo, Japan; 3 Department of Clinical Pathology, and Department of Biomedical and Clinical Sciences, Linköping University, Linköping, Sweden; 4 Department of Statistics, Umeå School of Business Economics and Statistics, Umeå University, Umeå, Sweden; 5 Department of Surgery, University of Helsinki and Helsinki University Hospital, Helsinki, Finland; 6 Department of Surgery, Translational Cancer Medicine Research Program, Faculty of Medicine, University of Helsinki and Helsinki University Hospital, Helsinki, Finland; 7 Department of Transplantation Surgery, Institute of Clinical Sciences, Sahlgrenska Academy, University of Gothenburg and Transplant Institute, Sahlgrenska University Hospital, Gothenburg, Sweden; 8 Department of Diagnostics and Intervention, Oncology, and Wallenberg Centre for Molecular Medicine (WCMM), Umeå University, Umeå, Sweden; 9 Department of Oncology, and Department of Biomedical and Clinical Sciences, Linköping University, Linköping, Sweden

**Keywords:** Pancreatic ductal adenocarcinoma, CLDN18.2, Immunohistochemistry, Prognosis, Surgical resection

## Abstract

**Background:**

Targeting Claudin-18 isoform 2 (CLDN18.2) improves survival in CLDN18.2 positive advanced gastric cancer and similar trials on metastatic pancreatic ductal adenocarcinoma (PDAC) are ongoing. Here, we aimed to assess the prevalence of CLDN18.2 positivity in resected PDAC including its association with overall survival (OS).

**Methods:**

Immunohistochemical analysis on paraffin-embedded primary tumors and lymph node metastases was performed from patients who underwent curative-intent PDAC resection at four centers in Sweden and Finland. In addition, survival outcomes were assessed.

**Results:**

In total, 599 primary tumors and 197 lymph nodes were analyzed. CLDN18.2 positivity was observed in 138 of 599 of primary tumors (23%) and 35 of 197 metastatic lymph nodes (17.8%). CLDN18.2 positivity in primary tumors was associated with higher tumor grade (*p* = 0.016). Median OS was longer in patients with CLDN18.2-positive tumors (27.7 vs. 21.1 months). Adjusted analysis identified CLDN18.2 positivity as an independent prognostic variable for OS (hazard ratio 0.79; 95% confidence interval 0.64–0.98).

**Conclusions:**

CLDN18.2 is expressed in 23% of primary PDAC tumours, indicating its potential to be used as a therapeutic target in a significant proportion of PDAC patients, and its expression is associated with improved OS in resected PDAC, supporting its role as a prognostic marker.

**Supplementary Information:**

The online version contains supplementary material available at https://doi.org/10.1245/s10434-026-20191-5.

Pancreatic ductal adenocarcinoma (PDAC) is the third-leading cause of cancer-related death and is expected to become the second-leading cause by 2030.^1,2^ Approximately 20% of patients with PDAC are eligible for surgical resection, which is the only potentially curative treatment. Postoperative chemotherapy is essential to reduce the risk of relapse in primarily resectable PDAC. Similarly, preoperative chemo(radio)therapy has a key role in borderline resectable or locally advanced disease.^3,4^ Although advances in chemotherapy, such as FOLFIRINOX, gemcitabine plus nab-paclitaxel, and gemcitabine plus capecitabine, have improved outcomes, the prognosis remains poor for most patients.^5–7^ No effective precision medicine strategies have yet been established for PDAC, highlighting the urgent need for molecularly targeted or biomarker driven therapies.^8^

Claudin-18 isoform 2 (CLDN18.2) is involved in the formation of tight junctions between epithelial cells and is highly expressed in normal gastric epithelium and gastric cancer,^9^ where it contributes to epithelial cell adhesion and tumor microenvironment regulation.^10^ Recently, the SPOTLIGHT randomized phase 3 trial demonstrated the efficacy of targeting CLDN18.2 in locally advanced unresectable or metastatic gastric or gastro-esophageal junction adenocarcinoma. In the study, Zolbetuximab, a monoclonal antibody targeting CLDN18.2, was used.^11^ Its role in treatment of PDAC remains unclear, but an ongoing phase II trial is evaluating CLDN18.2 targeted therapy in metastatic pancreatic cancer (NCT03816163). Previous studies have reported a wide range of CLDN18.2 expression in PDAC, from 36.9 to 56.5%.^13–15^ Zhang et al. indicates better overall survival (OS) in patients with CLDN18.2-positive tumors,^12^ while others report no significant difference.^13,14^

This study was designed to assess the prevalence of CLDN18.2 expression in primary tumors and lymph nodes from patients with PDAC and to examine clinicopathological and survival associations with CLDN18.2 expression.

## Methods

### Study Design and Patients

This international multicenter study included patients who underwent curative intent resection for pancreatic ductal adenocarcinoma (PDAC) at three Swedish university hospitals: Umeå University Hospital; Linköping University Hospital and Sahlgrenska University Hospital; as well as Helsinki University Hospital, Finland, between 1993 and 2020. This whole cohort included both upfront operated and neoadjuvant treated therapy. Clinicopathological data were retrospectively collected. TNM stage was classified according to the AJCC/UICC 8th edition.^15^ According to the WHO classification, tumors were graded as well differentiated, moderately differentiated, or poorly differentiated, based on histological features.^16^ Margin status was classified according to local pathological assessment. R0 resection was defined as no tumor cells within 1 mm of any resection margin, R1 resection as the presence of tumor cells within 1 mm of a resection margin, and R2 resection as macroscopic residual disease.^17^ Clinicopathological data from the patients and the results of immunohistochemical staining were used for the analysis. This study was approved by the Swedish Ethical Review Authority and the Helsinki University Hospital Ethics committee (DNR: 2021-02787, 2020-01511, 09‐175M/2009‐1378‐31, HUS/1223/2021). This study was conducted in accordance with the ethical principles of the Declaration of Helsinki.

### Tissue Microarray

Formalin-fixed, paraffin-embedded (FFPE) PDAC tumor and lymph nodes specimens were sectioned and allocated to tissue microarray (TMA) blocks. Representative tissue areas were selected from tumor regions identified by an experienced pancreatic pathologist at each institution using hematoxylin and eosin (H&E)-stained sections. Tissue microarrays (TMAs) were constructed with two to three cores (1 mm in diameter) taken from each primary tumor block, resulting in four to seven cores per patient. Additionally, if lymph node metastases were present, one core was taken from each metastatic block, with one to two cores per patient included in the TMA slides. Subsequent sections of the TMA blocks were cut at 5-µm thickness and mounted onto glass slides for immunohistochemical staining. The construction and Swedish part of the cohort has been previously described.^18^

### Immunohistochemistry

CLDN18.2 protein expression was stained using an Anti-Claudin 18 antibody (clone 43-14A, Abcam, Cambridge, UK) at a dilution of 1:200 using the Ventana BenchMark ULTRA system according to the protocol described in the manufacturer’s package insert. The antibody clone 43-14A used in this study may not exclusively distinguish between the CLDN18.1 and CLDN18.2 isoforms. However, CLDN18.1 expression is mainly restricted to normal lung tissue, whereas CLDN18.2 expression is largely confined to normal gastric mucosa and is frequently retained in gastric cancer and ectopically expressed in pancreatic ductal adenocarcinoma.^19^ Counterstaining was performed by using H&E.

### Scoring of CLDN18.2 Protein Expression

**Fig. 1:**
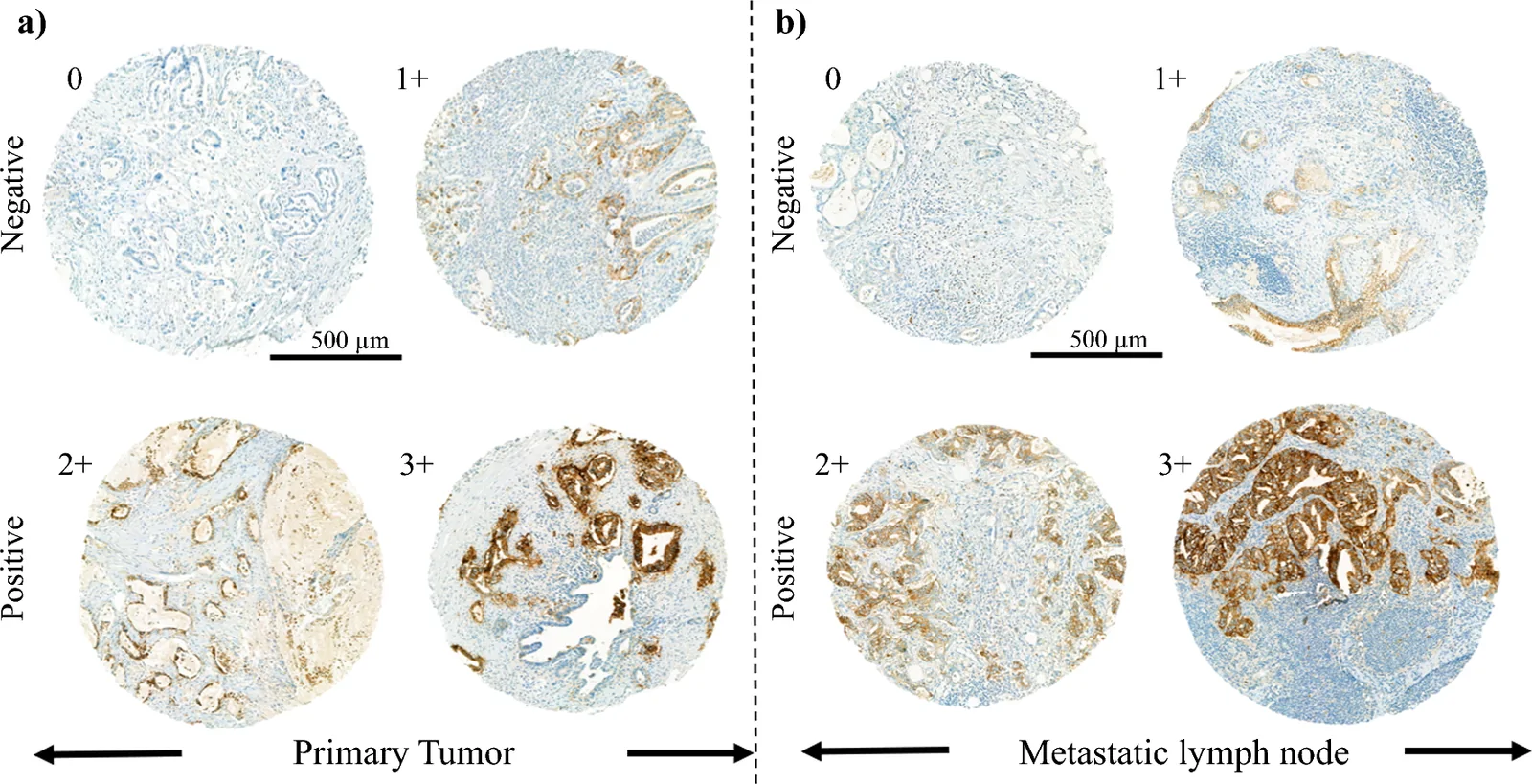
(**a**) Immunohistochemical staining of pancreatic cancer tissue microarray. CLDN18.2 expression intensity was categorized into 0, 1+, 2+, and 3+. (**b**) Immunohistochemical staining of metastatic lymph nodes from pancreatic cancer tissue microarray. CLDN18.2 expression intensity was categorized into 0, 1+, 2+, and 3+.

The CLDN18.2 expression intensity in the TMA samples was microscopically analyzed and scored by two experienced pathologists (H.B. and S.F.), and any differences in assessment of staining were resolved by discussing and reviewing the sample. Both pathologists were blinded to the clinical data. The expression was scored both in terms of staining intensity and distribution. Based on previous studies,^11,20^ the staining intensity of CLDN18.2 was scored into the following four categories: 0 = no membranous staining; 1+ = faint to < 75% moderate membranous staining; 2+ = ≥ 75% moderate membranous staining; and 3+ = ≥ 75% strong membranous staining (Fig. 1). A case was considered positive if at least two TMA cores were evaluable and at least one core exhibited a staining intensity of 2+ or higher. For metastatic lymph nodes, a similar scoring system was applied; however, a case was considered positive if at least one TMA core was evaluable and had a staining intensity of 2+ or higher.

### Statistical Analysis

Statistical analyses were performed using SPSS version 29 (IBM Corp., Armonk, NY). Independent two-sided *t*-test was used to compare mean values between two groups. Categorical variables were analyzed using the chi-square test (*χ*^2^ test). Additionally, the Phi coefficient was calculated to assess the strength of association between two binary variables, particularly for the relationship between CLDN18.2 positivity in primary tumors and in metastatic lymph nodes.^21^ For survival analysis, Kaplan-Meier curves were generated, and differences between survival curves were assessed by using the log-rank test. Factors influencing survival were further analyzed by Cox proportional hazards model to identify independent prognostic variables. A *p*-value < 0.05 was considered statistically significant throughout analyses. Multiple imputation (MI) was applied for the Cox proportional hazards model to handle missing data under the assumption that the data were missing at random. The MI procedure used chained equations (MICE) to generate 20 imputed datasets. The imputation model included following variables: age, sex, histological grade, T status (UICC 8th edition^15^), the presence of metastatic lymph nodes, neoadjuvant chemotherapy, margin status, and CLDN18.2 positivity, as well as the Nelson-Aalen estimator of the cumulative hazard and a death event indicator.^22^ Only T status from the TNM staging system was included in the imputation model and clinical stage and N status (UICC 8th edition) were excluded owing to more than 30% missing data, which could compromise the reliability of the imputation. The variables imputed using MI were subsequently utilized in evaluating both unadjusted and adjusted associations with prognosis.

### Intratumoral Heterogeneity Analysis

Intratumoral heterogeneity was evaluated using multiple TMA cores obtained from each tumor. Each core was scored individually according to the CLDN18.2 staining intensity scoring system described above, using the four‑level staining intensity scale (0, 1+, 2+, 3+). Tumors were classified as “low” when all cores showed low scores (0, 1+), “high” when all cores showed high scores (2+, 3+), and “mixed” when both low and high scores were present within the same tumor. The distribution of tumor classification was analyzed according to the number of evaluated cores. Differences in tumor classification according to the number of evaluable cores were assessed using the chi-square test. Agreement between cores within the same tumor was assessed by using Krippendorff’s alpha for ordinal data, computed on the original four‑level staining intensity scores, which allows for variable numbers of observations per case.^23^ Ninety‑five percent confidence intervals were estimated by using bootstrap resampling.

## Results

### Study Cohort

**Fig. 2:**
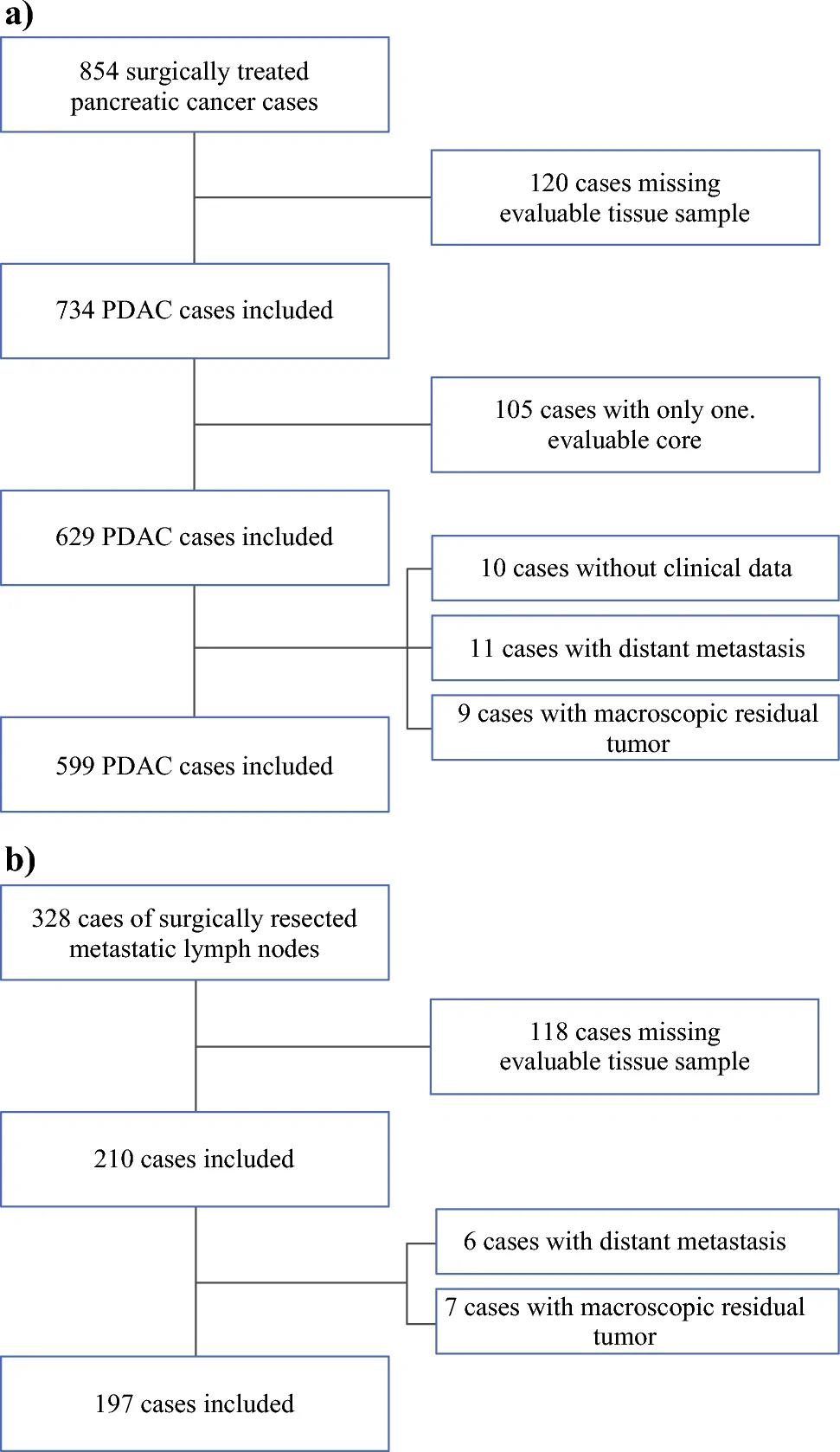
(**a**) The inclusion and exclusion of patients in the TMA cohort of primary tumors. In total, 854 surgically treated patients with histologically confirmed diagnosis of PDAC were included in the study. Patients with at least two evaluable cores are included. (**b**) The inclusion and exclusion of patients in the TMA cohort of lymph node metastases. In total, 328 cases with surgically resected metastatic lymph nodes of PDAC were included. Patients with at least one evaluable core were included.

We performed immunohistochemical staining on primary pancreatic tumors from 854 patients and metastatic lymph nodes from 328 patients. The distribution of patients with resected primary pancreatic tumors and metastatic lymph nodes across the four participating centers is shown in Supplementary Table 1. Among the 854 patients, a total of 120 samples were not evaluable due to incomplete TMA cores or insufficient tumor areas for assessment (Fig. 2a). Following the exclusion of these cases and 105 other patients with only one evaluable core, 629 patients with at least two evaluable TMA cores and available clinical data remained. Of these, 11 with distant metastases and nine with R2 resections were excluded, leaving 599 patients for final analysis. Lymph node analysis included 197 evaluable cases without distant metastases or R2 resections (Fig. 2b). Table 1 presents a comparison of clinicopathological characteristics between patients with CLDN18.2-positive and CLDN18.2-negative tumors. Tumor grade was higher in CLDN18.2 positive patients (*p* = 0.016). There were no statistically significant differences regarding age, sex, clinical stage, pathological *T* stage, the rate of regional lymph node metastasis, the rate of margin status, or the use of neoadjuvant chemotherapy.

**Table 1:** Clinical characteristics of CLDN18.2 positive and negative patients

	Total	CLDN18.2 positive	CLDN18.2 negative	*p*
Parameters	*N* = 599	*n* = 138	*n* = 461	
Age, *n* (%)				
≥ 65	395	98 (72.2%)	297 (65.5%)	0.18
< 65	204	40 (27.8%)	164 (34.5%)	
Sex, *n* (%)				
Male	262	58 (44.3%)	204 (46.9%)	0.62
Female	304	73 (55.7%)	231 (53.1%)	
Unknown	33	7	26	
Tumor grade				0.016
Well differentiated	55	20 (14.7%)	35 (7.9%)	
Moderately differentiated	319	78 (57.4%)	241 (54.2%)	
Poorly differentiated	207	38 (27.9%)	169 (38.0%)	
Not assessed	18	2	16	
Clinical stage, *n* (%) UICC 8th				0.9
IA, IB	90	25 (26.3%)	65 (22.9%)	
IIA, IIB	148	35 (36.8%)	113 (39.8%)	
III	102	26 (27.4%)	76 (26.7%)	
IV	39	9 (9.5%)	30 (10.6%)	
Unknown	220	43	177	
Pathological *T* stage, UICC8th				0.73
*T*, *n* (%)				
(y)pT1a, b, c	61	17 (15.9%)	44 (12.3%)	
(y)p T2	294	66 (61.7%)	228 (63.9%)	
(y)p T3	99	21 (19.6%)	78 (21.8%)	
(y)p T4	10	3 (2.8%)	7 (2.0%)	
Missing	135	31	104	
Pathological nodal staging, *n* (%)				0.22
(y)pN1-2	443	97 (70.3%)	346 (75.7%)	
(y)pN0	152	41 (29.7%)	111 (24.3%)	
Missing	4	0	4	
Margin status, *n* (%)				0.32
R0	368	80 (58.4%)	288 (63.4%)	
R1	223	57 (41.6%)	166 (36.6%)	
Missing	8	1	7	
Neoadjuvant chemotherapy, *n* (%)				0.88
Yes	69	15 (10.9%)	54 (11.7%)	
No	528	122 (89.1%)	406 (88.3%)	
Unknown	2	1	1	

### Prevalence of CLDN18.2 Expression in Primary Tumors and Lymph Node Metastases

Figure 1 presents representative immunohistochemical staining images of primary pancreatic tumors and metastatic lymph nodes. Among the 599 patients with evaluable primary tumors, 138 (23%) were classified as positive. Regarding metastatic lymph nodes, 35 of 197 cases (17.8%) were positive. CLDN18.2 expression in primary tumors were associated with expression in regional lymph node metastases (*p* < 0.001, Phi Coefficient 0.33) (Table 2).

**Table 2:** Comparison of CLDN18.2 expression in metastatic lymph nodes according to primary tumor CLDN18.2 status

	CLDN18.2 expression of metastatic Lymph node		
	Positive, *n*	Negative, *n*	Lymph node tested
CLDN18.2 expression of primary tumor			
Positive	22 (44%)	28 (56%)	50
Negative	13 (8.8%)	134 (91.2%)	147
Total	35	162	197

Chi-square test, *p* < 0.001, Phi coefficient was 0.33

### Association Between CLDN18.2 Expression and Prognosis

**Fig. 3:**
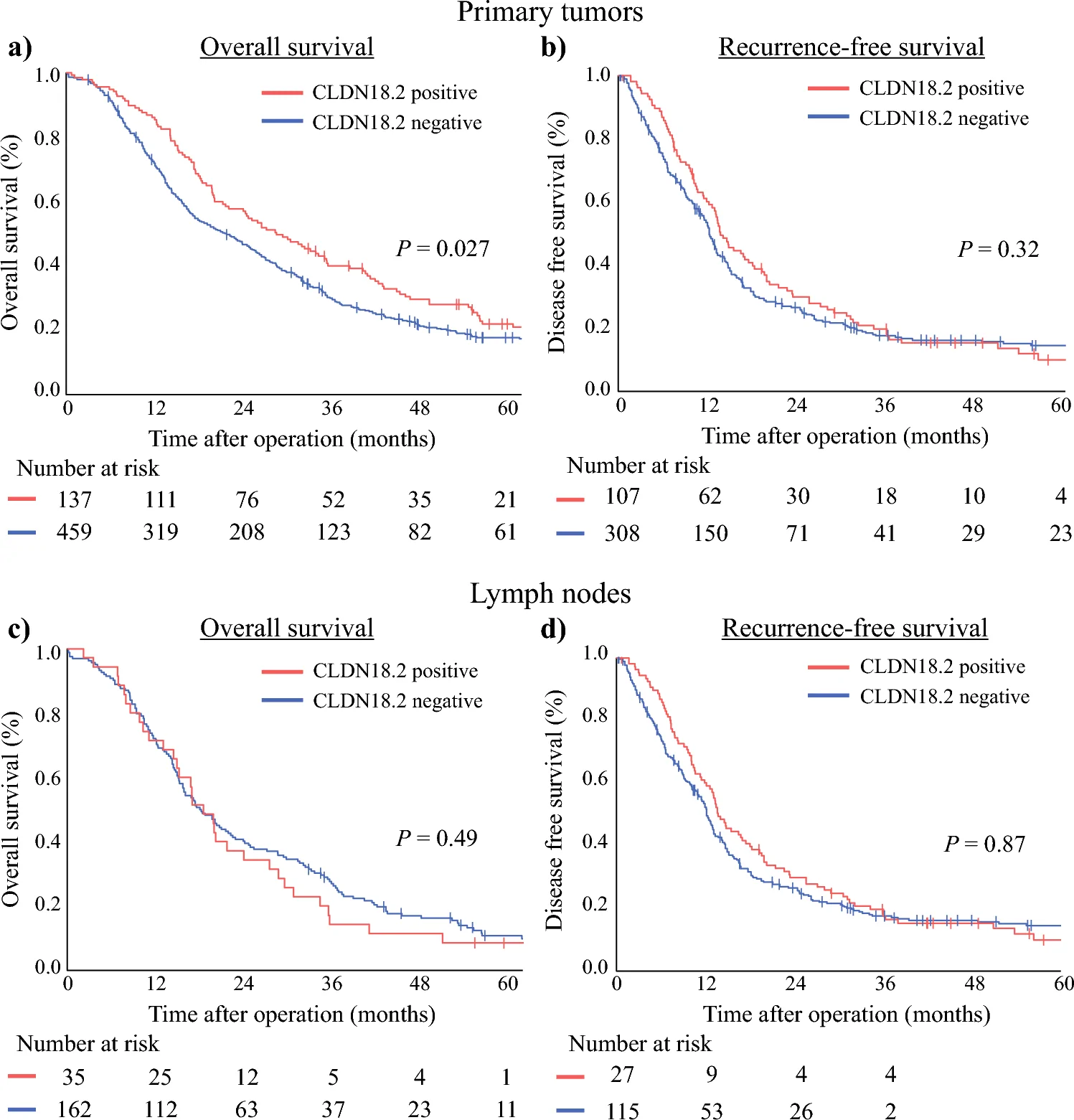
(**a**) Overall survival and (**b**) recurrence-free survival of patients with surgically treated pancreatic cancer according to CLDN18.2 expression. (**c**) Overall survival and (**d**) recurrence-free survival of patients with lymph node metastasis of pancreatic cancer according to CLDN18.2 expression

Figure 3 shows the association between CLDN18.2 expression and survival outcomes. Patients with CLDN18.2-positive primary tumors had better OS (median OS 27.7 months [95% confidence interval (CI) 21.3–34.1] vs. 21.1 months [95% CI 17.6–24.5]). The difference in survival distributions was statistically significant (*p* = 0.027, log-rank test). In contrast, recurrence-free survival (RFS) was similar between the two groups. CLDN18.2 staining in lymph nodes was not associated with OS or RFS (Fig. 3c, d).

In unadjusted Cox regression (Table 3), CLDN18.2 positivity was associated with better OS (hazard ratio [HR] 0.80; 95% CI 0.65–0.98; *p* = 0.032). In the adjusted Cox regression model, the following variables were independently associated with OS: histological grade, T3 or T4 stage, lymph node metastasis, neoadjuvant chemotherapy, margin status, and CLDN18.2 positivity in the primary tumor (HR 0.79; 95% CI 0.64–0.98; *p* = 0.034) (Table 3). CLDN18.2 expression was not associated with RFS (Supplementary Table 2). Since samples dated back to 1990s, we performed a sensitivity analysis excluding samples from before 2010 (*n* = 447 remained in the analysis). Adjusted Cox regression analysis in this contemporary cohort generated similar results (Supplementary Table 3).

**Table 3:** Unadjusted and adjusted Cox regression analysis for overall survival

Parameters	Overall survival			
	Unadjusted analysis		Adjusted analysis	
	HR (95% CI)	*p*	HR (95% CI)	*p*
Age ≥ 65 years	1.09 (0.91–1.31)	0.35	1.11 (0.91–1.33)	0.32
Male (vs. female)	1.13 (0.94–1.36)	0.18	1.11 (0.92–1.33)	0.28
Histological grade				
Poorly differentiated (vs. well and moderately differentiated)	1.40 (1.16–1.69)	< 0.001	1.22 (1.00–1.48)	0.049
*T* Stage, UICC8th				
T3, 4 (vs. T1, T2)	1.48 (1.17–1.88)	0.001	1.38 (1.09–1.75)	0.008
Regional nodal metastasis	1.76 (1.43–2.18)	< 0.001	1.62 (1.30–2.01)	< 0.001
Neoadjuvant chemotherapy	0.68 (0.51–0.91)	0.009	0.72 (0.54–0.97)	0.029
Margin status R1 (vs. R0)	1.48 (1.23–1.77)	< 0.001	1.31 (1.08–1.59)	0.006
Claudin18.2-positive (vs. negative)	0.80 (0.65–0.98)	0.032	0.79 (0.64–0.98)	0.034

During follow-up, 505 deaths occurred and were included in the multivariable Cox proportional hazards analysis

### Sensitivity Analysis According to the Neoadjuvant Treatment Status

Neoadjuvant treated patients and upfront surgery treated patients were analysed separately in sensitivity analyses. In the neoadjuvant-treated group, CLDN18.2 positivity was not associated with either OS or RFS (Supplementary Table 4). In contrast, in the upfront surgery group, CLDN18.2 positivity was associated with significantly better OS but not with RFS.

### Intratumoral Heterogeneity of CLDN18.2 Expression

Intratumoral heterogeneity was evaluated using multiple TMA cores per tumor. A subset of tumors showed mixed expression patterns across cores. The distribution of low, mixed, and high tumors differed significantly according to the number of evaluable cores (*p* < 0.001), with the proportion of mixed tumors increasing from 12.1 and 11.5% in tumors with two and three evaluable cores to 25.8% in tumors with four to six evaluable cores (Supplementary Fig. S1). Inter-core agreement was moderate (Krippendorff’s *α* = 0.59, 95% CI 0.54–0.61).

## Discussion

Targeting CLDN18.2 in gastrointestinal cancers is a novel and promising approach. Here, we analyzed the prevalence of CLDN18.2 in resected pancreatic cancer primary tumors and lymph node metastases as well as exploring its prognostic value in a large international multicenter cohort. We demonstrate that the prevalence of CLDN18.2-positivity in primary tumors is 23% (138/599) and that CLDN18.2 expression in the primary tumor is associated with well differentiated tumor grade and expression in regional lymph node metastases. Furthermore, CLDN18.2-positivity is associated with better OS.

Our findings suggest that almost one in four pancreatic cancer patients may be eligible for CLDN18.2-targeted therapy, a finding with potentially considerable clinical implications. The SPOTLIGHT trial in gastric and gastroesophageal junction cancers demonstrated a survival benefit in CLDN18.2-positive patients when treated with the monoclonal antibody Zolbetuximab. Zolbetuximab has been tested in human pancreatic cancer in vitro^24^ and suggests antibody-dependent cellular cytotoxicity (ADCC) and complement-dependent cytotoxicity (CDC) effects on pancreatic cancer (PC) cells. Additionally, Zolbetuximab has antitumor activity in cell-derived mouse xenograft models of pancreatic cancer. Interestingly, the cytotoxic effects correlate with CLDN18.2 expression on cell surfaces.^24^ There is an ongoing phase II study assessing the safety and efficacy of gemcitabine and nab-paclitaxel (GN) alone versus GN combined with Zolbetuximab in CLDN18.2-positive metastatic PDAC (NCT03816163). Although the study is focused on metastatic pancreatic cancer, our results raise the possibility that CLDN18.2-targeted therapies may also have a role in the perioperative setting. Furthermore, we observed that patients with CLDN18.2-positive primary tumors were more likely to have CLDN18.2-positive lymph node metastases. Similar to our findings, Wöll et al. demonstrated that CLDN18.2 expression is generally maintained in metastatic lymph nodes of PDAC patients.^25^ While CLDN18.2 is expected to be absent in non-metastatic lymph nodes, 8.8% of patients in our cohort showed discordant expression, with negative primary tumors but positive metastatic lymph nodes (Table 2). This suggests intratumoral heterogeneity and indicates that evaluation of metastatic sites may provide additional information beyond the primary tumor. Lymph node status is one of the strongest prognostic factors in PDAC, and therapies that effectively target metastatic lymph nodes may improve patient outcomes (Table 3). This finding suggests the possibility of targeting metastatic nodes and a potential efficacy of CLDN18.2 targeted therapies.

The prevalence of CLDN18.2 positivity in our cohort is relatively low compared with previous reports. This is possibly explained by (1) differences in study cohort size, (2) differences in staining protocols, and (3) selections of cut-off levels to define CLDN18.2 positivity. While our study shares similarities with the publication by Arseneau RJ, there are several important differences.^26^ First, our study is a multicenter study with a larger sample size. Second, our study was specifically designed to evaluate intratumoral heterogeneity using quantitative methods, whereas Arseneau et al. assessed CLDN18 expression based on the proportion of positive tumor cells and staining intensity. We present data on the largest cohort so far, using the same antibody clone and the same cutoffs as utilized in the published SPOTLIGHT trial as well as in ongoing trials. Zhang et al. reported that 56.5% of 302 PDAC samples were positive (antibody: Wuxi Diagnostics, cutoff: 1+, 1% of cells). They defined a broader range of positivity with 1+ to 3+ cases as positive. Park et al. reported that 41 of 130 patients (31.5%) were positive (antibody: Invitrogen, cutoff: 2+, 80% of cells); however, given the relatively small sample size, sampling bias in the results cannot be ruled out.^13^ In gastric cancer studies, the reported prevalence of CLDN18.2 positivity also varies among studies. The FAST study reported a prevalence of 49% (334/686) using the CLAUDETECTTM18.2 histology kit (cutoff: 2+ in 40% of cells),^27^ the ILUSTRO study found a prevalence of 46.2% (162/351) with an unknown antibody (cutoff: 2+ in 50% of cells),^20^ and the SPOTLIGHT study reported 38% (922/2403) using the VENTANA CLDN18 [43-14A] RxDx Assay (cutoff: 2+ in 75% of cells).^11^ In our study, the intensity cutoff was determined primarily based on the SPOTLIGHT trial. To account for tumor heterogeneity, only patients with two evaluable cores were included in the analysis.

CLDN18.2-positive tumors were significantly associated with well to moderately differentiated tumors.^12,13,25^ In contrast, in gastric cancer, tumor grade did not differ significantly between CLDN18.2-positive and CLDN18.2-negative patients.^28,29^ While the exact mechanism remains to be fully elucidated, emerging evidence suggests that CLDN18 is regulated by PKC and MAPK signaling pathways, thereby contributing to tumor-related signaling and tumor progression.^10,30^ In addition, recent evidence indicates that oncogenic KRAS signaling, particularly KRASG12D, may drive O-GlcNAcylation of CLDN18.2, thereby altering its cellular localization, enhancing pancreatic cancer progression, and reducing the efficacy of CLDN18.2-targeted therapy.^31^ These findings suggest that CLDN18.2 is not merely a structural tight junction protein but may reflect underlying tumor biology.

Patients with CLDN18.2-positive primary tumors had better OS and CLDN18.2-positivity was an independent prognostic marker in adjusted analysis. These findings suggest that CLDN18.2 expression may be a prognostic marker for PDAC. The prognostic significance of CLDN18.2 was preserved in a contemporary cohort, supporting its robustness across treatment eras. Importantly, CLDN18.2 should be interpreted as a prognostic rather than a predictive biomarker in the present study, as patients in our cohort were not treated with CLDN18.2-targeted therapies. An ongoing phase II trial is evaluating zolbetuximab in CLDN18.2-positive metastatic PDAC (NCT03816163), which may help to clarify its predictive role.

Regarding the prognostic impact, Zhang et al. observed that strong, diffuse CLDN18.2 immunoreactivity was associated with significantly prolonged OS in resected PDAC,^12^ while RFS was largely unchanged. Similarly, in our cohort, CLDN18.2 positivity was associated with improved OS but not RFS. The reason for this discrepancy remains unclear. It is possible that CLDN18.2 expression reflects aspects of tumor biology that influence overall survival beyond recurrence risk alone. Further studies are needed to clarify the biological mechanisms underlying this observation. Zhang et al. further reported that CLDN18.2 tended to be lost in the EMT phenotype, which may explain why CLDN18.2-positive patients showed better survival, although the detailed molecular mechanism has not been fully understood.^12^ In contrast, other cohorts have not demonstrated a survival benefit: Park et al. and Valentini et al. each reported no significant differences in either overall or recurrence-free survival between CLDN18.2-positive and -negative patient groups.^13,14^ Notably, all these studies including ours, consistently found CLDN18.2 overexpression to be more frequent in well or moderately differentiated tumors, and some also reported an association with node-negative tumors.^12–14^ Such favorable clinicopathologic associations could contribute to the improved outcomes seen in CLDN18.2-high subsets. Taken together, the results suggest that CLDN18.2 may hold potential prognostic relevance, although its impact on survival has not been uniformly observed across studies. The differing results underscore the need for further large-scale validation to clarify the prognostic role of CLDN18.2 in PDAC. In the neoadjuvant subgroup, CLDN18.2 positivity was not significantly associated with OS or RFS. Given the relatively small number of patients in this subgroup, this lack of statistical significance may reflect limited statistical power. It is also possible that CLDN18.2 tissue expression changes during neoadjuvant therapy or that patient selection following neoadjuvant therapy impacted the results.

Intratumoral heterogeneity is an inherent challenge in tissue microarray-based biomarker studies. By evaluating multiple cores per tumor, we observed mixed expression patterns in a subset of cases, with heterogeneity more frequently detected as the number of evaluated cores increased. These findings suggest that intratumoral heterogeneity reflects underlying tumor biology rather than random variation and should be considered when interpreting CLDN18.2 expression in PDAC. Conversely, even evaluation of larger tissue sections represents a small proportion of the tumor. Importantly, we propose that scoring systems for CLDN18.2 should account for the highest score found within evaluated samples, rather than analyzing an average score across scores or a tissue.

Despite the novel findings, our study has several limitations. First, it is a retrospective study and might be subject to potential biases. Second, a substantial part of our cohort underwent surgery without neoadjuvant chemotherapy. As a result, we were unable to analyze the potential influence of these combination chemotherapies on survival outcomes. Third, nonmetastatic lymph nodes were not systematically assessed, which precludes a comprehensive evaluation of CLDN18.2 expression across all nodal tissues.

Nonetheless, our study also has important strengths. It is based on a large, international, multicenter cohort with robust clinicopathological data. Additionally, our cohort reflects a real-world, population-based setting from Sweden and Finland, where health care is publicly funded and universally accessible, ensuring inclusion of patients irrespective of socioeconomic status. We used a validated anti-CLDN18.2 antibody and a clinically relevant and standardized scoring system. Further studies are warranted to validate CLDN18.2 expression using complementary methods and to clarify its biological and clinical significance.

In addition, prospective clinical trials will be needed to determine whether CLDN18.2 can guide patient selection for targeted therapies in PDAC.

## Conclusions

Our study demonstrates that CLDN18.2 is expressed in 23% of resected pancreatic ductal adenocarcinomas and that its expression in primary tumors is associated with better OS. These findings suggest that CLDN18.2 may serve as both a prognostic biomarker and a potential therapeutic target for a significant proportion of PDAC patients. Given the successful application of CLDN18.2-targeted therapies in gastric cancer, further clinical studies are warranted to evaluate their efficacy in pancreatic cancer and role in perioperative therapy, particularly in patients with high CLDN18.2 expression.

## Supplementary Information

Below is the link to the electronic supplementary material.

Supplementary file1 (XLSX 10 KB)

Supplementary file2 (XLSX 11 KB)

Supplementary file3 (XLSX 11 KB)

Supplementary file4 (XLSX 11 KB)

Supplementary file5 (TIF 1601 KB)

## Data Availability

The datasets analyzed in the current study are not publicly available due to patient confidentiality and ethical restrictions. De-identified data are available from the corresponding author upon reasonable request and approval from the relevant ethics committee.
